# Precision Colorectal Cancer Fecal Immunological Test Screening With Fecal-Hemoglobin-Concentration–Guided Interscreening Intervals

**DOI:** 10.1001/jamaoncol.2024.0961

**Published:** 2024-05-09

**Authors:** Amy Ming-Fang Yen, Chen-Yang Hsu, Ting-Yu Lin, Chiu-Wen Su, Han-Mo Chiu, Tony Hsiu-Hsi Chen, Sam Li-Sheng Chen

**Affiliations:** 1 School of Oral Hygiene, College of Oral Medicine, Taipei Medical University, Taipei, Taiwan; 2 Dachung Hospital, Miaoli, Taiwan; 3 Institute of Epidemiology and Preventive Medicine, College of Public Health, National Taiwan University, Taipei, Taiwan; 4 Department of Internal Medicine, National Taiwan University Hospital, Taipei, Taiwan; 5 Department of Internal Medicine, College of Medicine, National Taiwan University, Taipei, Taiwan; 6 TMU Research Center of Cancer Translational Medicine, Taipei Medical University, Taipei, Taiwan; 7 TMU Research Center for Digestive Medicine, Taipei Medical University, Taipei, Taiwan

## Abstract

**Importance:**

Given a gradient relationship between fecal hemoglobin (f-Hb) concentration and colorectal neoplasia demonstrated previously, using f-Hb–guided interscreening interval has increasingly gained attention in population-based fecal immunological test (FIT), but it is very rare to address how to implement such a precision strategy and whether it can economize the use of FIT and colonoscopy.

**Objective:**

To demonstrate the applicability of personalized colorectal cancer (CRC) screening with f-Hb–guided screening intervals to reduce the number of FITs and colonoscopy with as equivalent efficacy as universal biennial screening.

**Design, Setting, and Participants:**

A retrospective cohort study for developing f-Hb–guided precision interscreening interval was conducted using data on a Taiwanese biennial nationwide FIT screening program that enrolled more than 3 million participants aged 50 to 74 years between 2004 and 2014. The cohort was followed up over time until 2019 to ascertain colorectal neoplasia and causes of death. A comparative study was further designed to compare the use of FIT and colonoscopy between the personalized f-Hb–guided group and the universal biennial screening group given the equivalent efficacy of reducing CRC-related outcomes.

**Main Outcomes and Measurements:**

A spectrum of f-Hb–guided intervals was determined by using the Poisson regression model given the equivalent efficacy of a universal biennial screening. The use of FIT and colonoscopy for the pragmatic f-Hb-guided interval group was measured compared with the universal biennial screening group. Data analysis was performed from September 2022 to October 2023.

**Results:**

Using data from the 3 500 250 participants (mean [SD] age, 57.8 [6.0] years) enrolled in the Taiwanese biennial nationwide FIT screening program, an incremental increase in baseline f-Hb associated with colorectal neoplasia and CRC mortality consistently was observed. Participants with different f-Hb levels were classified into distinct risk categories. Various screening intervals by different f-Hb levels were recommended. Using the proposed f-Hb–guided screening intervals, it was found that the personalized method was imputed to reduce the number of FIT tests and colonoscopies by 49% and 28%, respectively, compared with the universal biennial screening.

**Conclusion and Relevance:**

The gradient relationship between f-Hb and colorectal neoplasia and CRC mortality was used to develop personalized FIT screening with f-Hb–guided screening intervals. Such a precision interscreening interval led to the reduced use of FIT test and colonoscopy without compromising the effectiveness of universal biennial screening.

## Key Points

**Question:**

Can the gradient relationship between fecal-hemoglobin (f-Hb) concentration and colorectal neoplasia and death from colorectal cancer (CRC) be applied to precision interscreening interval of population-based CRC screening?

**Findings:**

In this cohort study including data from 3 500 250 participants, increasing f-Hb levels indicated higher colorectal neoplasia and CRC mortality risk, allowing for stratification of risk groups. Screening intervals could be calibrated on the basis of f-Hb levels: shorter intervals for higher f-Hb, longer intervals for lower f-Hb; with personalized f-Hb-guided screening, fecal immunological test use decreased by 49%, and colonoscopies decreased by 28% compared with universal biennial screening.

**Meaning:**

Personalized f-Hb–guided screening showed potential advantages over universal biennial screening in the optimal allocation of heath care resources to population-based screening for CRC.

## Teaser

This cohort study examines the applicability of personalized colorectal cancer (CRC) screening with fecal-hemoglobin (f-Hb)-guided screening intervals to reduce the number of fecal immunological tests and colonoscopy, as well as determine if it demonstrates equivalent efficacy as universal biennial screening.

## Introduction

Over the course of recent decades, significant strides have been taken to ameliorate the menace of colorectal cancer (CRC), a malignant disease ranking as the third most widespread across the globe. This progress is attributed to the implementation of population-based screening programs geared toward the early detection of CRC, in tandem with advancements in therapeutic modalities. Among the array of instruments harnessed within these screening initiatives, the fecal immunochemical test (FIT), a prominent stool-based diagnostic tool, has emerged as a pivotal determinant in curbing CRC mortality rates.^1,2,3,4^

Notwithstanding the well-documented efficacy of CRC screening contributing to primary prevention of arresting incident CRC through the removal of precancerous adenoma by colonoscopy as well as secondary prevention in mitigating CRC-linked fatalities through early detection of CRC, a pivotal facet has thus far evaded comprehensive investigation: the judicious exploitation of fecal-based biomarkers to classify the populace into discrete strata of CRC susceptibility. Similar to a risk-based screening for other cancers such as breast cancer with a focus on breast density proposed before,^5^ such stratification holds promise in facilitating the tailoring of screening programs, rendering them more efficacious and resource-efficient, by tailoring interventions to individualized risk profiles.

The recent advent of quantitative FIT, targeting the quantification of hemoglobin concentration in stool specimens, has engendered new vistas of opportunity.^6^ Antecedent inquiries have unveiled notable disparities in fecal hemoglobin concentration (f-Hb) predicated on sex and age, thereby auguring the prospective utility of this biomarker in risk stratification endeavors.^7,8,9^ Association between elevated f-Hb levels and the magnitude and severity of colorectal neoplastic entities has also been revealed.^10,11,12^

Several studies using data from population-based CRC screening with FIT demonstrated a gradient relationship between f-Hb concentrations and the incidence of CRC, colorectal neoplasms, and CRC mortality.^13,14,15,16,17^ Such empirical findings underscore the potential of fine-tuning the use of quantitative f-Hb measurements as a means to devise individualized interscreening intervals for FIT-based CRC screening regimens. The temporal spacing of screening endeavors could be tailored commensurate with f-Hb concentrations: heightened levels prescribing abbreviated intervals, and conversely. Consequently, we posit that the integration of an f-Hb-guided framework for interscreening intervals has the potential to not only curtail the incidence of advanced CRC cases and concomitant mortalities, but also to alleviate the burden associated with colonoscopy, by rationalizing the volume of FIT tests and colonoscopies.

To actualize the aspiration of personalized interscreening intervals informed by f-Hb measurements, we aimed to comprehensively assess the association of f-Hb levels with the risk of adenoma, incident CRC, advanced CRC manifestations, and CRC-associated mortality. This endeavor was undertaken through a meticulous analysis of data derived from a nationwide cohort, participants of a FIT-based screening program, who were subject to screen over a median duration of 7.6 years in Taiwan. Our aim was to deduce recommendations for finely tuned interscreening intervals, orchestrated in accordance with f-Hb metrics. A comparative appraisal, pitting the universal biennial screening cohort against the f-Hb–guided counterpart, was subsequently undertaken to quantify the potential reduction in the quantum of FIT tests and colonoscopies, consequent to the strategic implementation of precision interscreening intervals.

## Material and Methods

### Study Design and Population

A retrospective cohort study was designed to first assess the gradient impact of f-Hb concentration on the risk of incidence and mortality rates of CRC. Specifically, the incremental concentration of f-Hb was categorized into 7 groups: undetectable, 1 to 9, 10 to 19, 20 to 49, 50 to 99, 100 to 149, and 150 or more μg Hb/g. The precision interscreening intervals were further recommended in the light of the gradient relationships between the incremental f-Hb categories and 3 outcomes of interest compared with the average risk of the underlying population.

The third step is to conduct a comparative design for the comparison of utilizations in FIT and colonoscopies between universal biennial screening and personalized f-Hb–guided screening following the programmatic interscreening interval given an equivalent reduction of CRC-related event-based outcomes including interval cancer, advanced CRC, and CRC death.

The data used for this study developing pragmatic precision interscreening interval by f-Hb categories were sourced from the Taiwanese Colorectal Cancer Screening Program, targeting individuals aged 50 to 74 years residing in Taiwan. Between 2004 and 2014, a substantial cohort of more than 3 million eligible Taiwanese residents (n = 3 500 250) actively participated in the nationwide biennial FIT screening program. In this analysis, a total of 3 487 559 cohort participants with complete f-Hb data were included from the pool of attendees. The institutional review board at Taipei Medical University approved this study (N202203051). The requirement for written informed consent was waived due to the use of deidentified data in accordance with regulation of the institutional review board.

### Procedure of Sample Collection

The FIT tests were supplied by 2 manufacturers: OC-Sensor (Eiken Chemical Company Ltd) and HM-JACK (Kyowa Medex Company Ltd). As per standard procedure, participants were provided with collection kits and directed to collect samples at home, subsequently returning them to the laboratory within a designated time frame.^1,18^ No specific dietary or medication restrictions were imposed. The collection device consisted of a brush probe holding approximately 10 mg and 0.5 mg of feces for OC-sensor and HM-jack, respectively, along with 2 mL of hemoglobin stabilization buffer.

### Data Collection

Demographic information including age, sex, screening date, and quantitative f-Hb results were extracted from a central screening database. Individuals whose baseline or repeated f-Hb readings exceeded the predefined cutoff value (20 μg Hb/g) were recommended for colonoscopy to ascertain colorectal neoplasia. The cohort was tracked until 2019 to identify incident colorectal neoplasia and death from CRC. In addition, the detailed individual data encompassing age, sex, screening date, residence, confirmatory diagnosis results, and detection of adenoma and CRC were gathered. Colorectal cancers were staged according to the *American Joint Committee on Cancer (AJCC) Staging Manual, eighth edition* guidelines, with stages 1 and 2 classified as early-stage CRC and stages 3 and 4 as advanced-stage CRC. A comprehensive account of the study’s design, screening protocol, referral and diagnostic policies, surveillance, and outcome assessment can be found elsewhere.^1,4^

### Statistical Analysis

We used a Poisson regression model to establish the association between incremental f-Hb levels and CRC mortality. The similar procedure was applied to interval cancer and advanced CRC. The model included a spectrum of risk profiles for CRC mortality in relation to the differential f-Hb levels as represented by the equation:



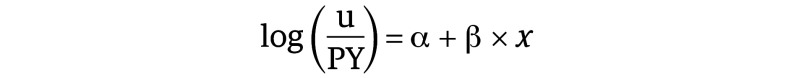



where *u* denotes the number of deaths from CRC and PY represents person-years. The variable *x* corresponds to the f-Hb category, whereas α signifies the CRC mortality of the reference group when *x* equals 0. The Poisson regression model estimates the coefficient β, which captures the relationship between the f-Hb category (0 = 20-49 μg Hb/g, 1 = other group) and the number of CRC deaths.

The equation from the previous expression could be written as:

log(u) = α + βx + log (PY)

The person-years (PY) can be calculated as *n* × 2 where *n* represents the number of individuals in each f-Hb group with a 2-year screening interval as the follow-up time. Consequently,

log(u) = α + βx + log (n × 2)

For *x* = 0 (f-Hb: 20-49 μg Hb/g), the equation is simplified to:

log(u_0_) = log(2n) + α

And for *x* = 1 (other f-Hb groups), the equation becomes:

log(u_1_) = log(kn) + α + β

where *k* denotes the recommended interscreening interval.

To equate *u_0_* = *u_1_* for the same population size

equation (1) – equation (2) log(*2n*) − *log*(*kn*) *− β = 0*

This leads to:



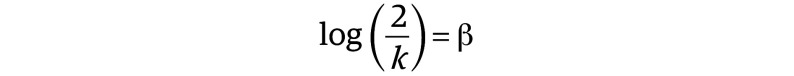



The value of *k* can then be obtained as:



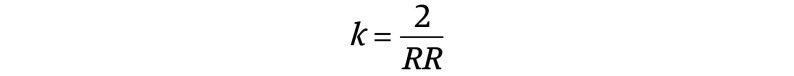



Theoretical precision interscreening intervals, contingent on f-Hb levels, were proposed by comparing each f-Hb–guided risk group to the average risk group, which is subjected to a biennial interscreening interval. Using the risk of average risk group as a reference to adjust the interscreening intervals for each f-Hb–guided risk group could reach the same performance for 2 types of screening. The Figure delineates a methodical comparative analysis of test and colonoscopy use. Both the biennial and personalized f-Hb–guided screening modalities manifest commensurate outcomes with respect to interval cancer rate, advanced cancer rate, or CRC mortality. To assess the practical implications of precision interscreening intervals determined by the Poisson regression model, the use of FIT test was imputed on the basis of empirical estimates of the baseline f-Hb categories identical to the first screen of biennial strategy among 3 487 559 participants. The use of colonoscopy among those with positive FIT results of those receiving FIT tests during the 12-year study period can be further imputed on the basis of the figures of the positive rate (8%), referral rate (80%), and adenoma detection rate (40%) identical to the universal biennial screening clinical data. The sensitivity analysis of pragmatic f-Hb–guided screening with various referral rates on the use of colonoscopy was conducted. We reported their potential to reduce the number of FIT tests and colonoscopies for the pragmatic f-Hb–guided screening group in comparison with the universal biennial screening group. All statistical analyses were conducted using SAS statistical software (version 9.4, SAS Institute). Data analysis was performed from September 2022 to October 2023.

**Figure.**
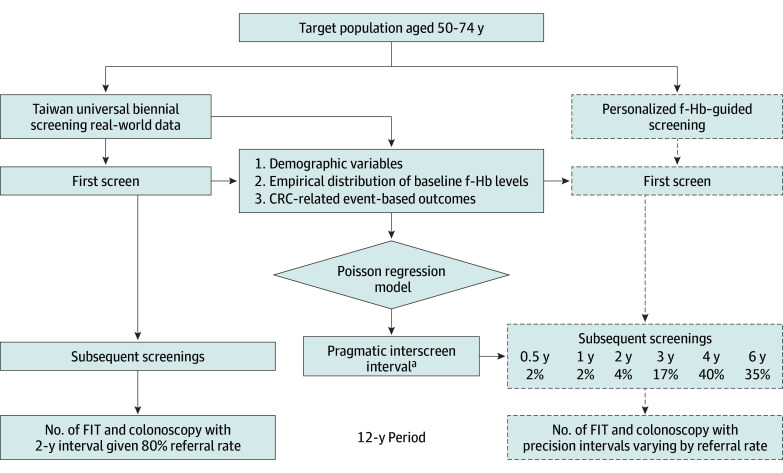
Comparative Analysis of Fecal Immunological Test (FIT) and Colonoscopy Use Between Biennial Screening and Personalized Fecal-Hemoglobin (f-Hb)-Guided Screening Given the Equivalent Efficacy Design The dashed lines indicate data on the basis of the f-Hb–guided personalized screening approach. CRC Indicates colorectal cancer. ^a^See Table 3.

## Results

### Gradient Relationships Between f-Hb Levels and CRC Outcomes

The rates of adenoma, CRC, advanced CRC incidence rates, and CRC mortality, by age, sex, and f-Hb level, are presented in Table 1. Notably, the rate of CRC incidence exhibited an ascending pattern with advancing age. A similar upward trajectory was also evident in the incidence of advanced CRC. The CRC mortality rate (per 100 000 people) increased from 10 for those aged 50 to 54 years to 53 for those aged 70 to 74 years. Male participants had the greater incidence rates and mortality than female participants. Intriguingly, a positive correlation between CRC incidence rates and f-Hb levels emerged. The CRC incidence rate (per 1000 person-years) increased with f-Hb, from 0.94 for undetected f-Hb to 10.25 for f-Hb 150 or more μg Hb/ g. eFigure 1 in Supplement 1 shows the cumulative rate of advanced CRC (per 1000 person-years) in a similar trend: 6.77 for those with undetectable f-HB, 8.68 for the 1 to 9 f-Hb range, 16 for 10 to 19, 16.3 for 20 to 49, 27.6 for 50 to 99, 18.7 for 100 to 149, and 45.2 for 150 or more μg Hb/g. This gradient trend is consistent with the findings presented in Table 1; eFigure 2 in Supplement 1, where a comparable upward trajectory was observed for mortality rates. The similar trend was also seen for colorectal adenoma as shown in Table 1.

**Table 1.** Number of Participants, Adenoma Cases, CRC Cases, Advanced CRC, Incidence Rates, and Mortality From CRC in a Population-Based Biennial FIT Screening Program

Characteristic	Participants, No.	Adenoma		CRC		Advanced CRC		CRC death	
		Incident cases	Incidence rate (per 1000)	Incident cases	Incidence rate (per 1000)	Incident cases	Incidence rate (per 1000)	Death	Mortality rate (per 100 000)
Age, y									
50-54	1 434 898	11 740	1.06	11 411	1.03	2898	0.26	1293	10
55-59	894 985	11 098	1.53	11 149	1.53	2860	0.39	1369	17
60-64	630 313	9727	1.91	10 196	1.99	2594	0.51	1453	25
65-69	452 596	5216	1.34	9026	2.31	2574	0.66	1842	42
70-74	87 458	47	0.11	1363	3.17	409	0.95	275	53
Sex									
Female	1 942 152	15 687	1.00	19 200	1.22	5416	0.34	2722	15
Male	1 558 098	22 141	1.85	23 945	1.99	5919	0.49	3510	26
f-Hb, μg Hb/g^a^									
Undetected	1 234 348	11 046	1.05	9952	0.94	2519	0.24	1318	11
1-9	1 394 140	15 396	1.38	15 511	1.38	3934	0.35	2124	17
10-19	580 536	5741	1.46	7264	1.85	1854	0.47	871	19
20-49	136 683	2305	2.33	3061	3.08	803	0.81	441	39
50-99	47 045	1008	3.01	1581	4.70	432	1.28	260	67
100-149	30 958	563	2.60	1012	4.67	277	1.28	164	66
≥150	63 849	1484	3.34	4494	10.25	1441	3.29	991	192
Total	3 500 250	37 828	1.36	43 145	1.55	11 335	0.41	6232	20

Abbreviations: CRC, colorectal cancer; f-Hb, fecal-hemoglobin; FIT, fecal immunological test.

^a^ f-Hb value was not available in 12 691 participants.

### Recommendations to f-Hb–Guided Interscreening Interval

Table 2 displays the predictive mortality rates for each f-Hb category. Participants with f-Hb between 20 and 49 μg Hb/g had a similar risk of CRC mortality as the general population (30.2 per 100 000). The increasing f-Hb in groups, which strongly suggests an increase in CRC mortality, can be used to stratify the underlying population into different risk groups to re-align the interscreening interval based on different f-Hb levels at baseline for reducing false-negative and false-positive results simultaneously. In Table 3, taking the 20 to 49 μg Hb/g category as the standard screening interval into account, participants with a higher f-Hb at the initial screening should undergo state-of-the-art confirmatory examinations with highly intensive surveillance, as their risk would be identical to that of the 20 to 49 μg/Hb g group with a biennial screening interval. On the other hand, the interval between repeated FIT screenings for those with a lower f-Hb could be lengthened to prevent false-positive results. For participants with f-Hb levels of 10 to 19 ug Hb/g and f-Hb levels of 1 to 9 ug Hb/g, respectively, the screening interval could be extended to 3 and 4 years. Similar findings were found if advanced cancer incidence was used as the comparison outcome (eTable 1 in Supplement 1). Similarly, the longer screening interval for the lower f-Hb levels would be suggested if interval cancer rate was used as the comparison outcome (eTable 2 in Supplement 1). It should be noted that theoretical recommendation based on risk-guided calculation was further simplified to pragmatic interscreening interval recommendation for feasibility.

**Table 2.** Predicted Mortality by f-Hb Using the Poisson Regression Model

Parameters	Regular coefficient	SD	Estimated mortality (per 100 000)	Mortality in general population (per 100 000)^a^
Intercept	−13.49	0.12	NA	NA
Age	0.08	0.002	NA	NA
Sex				
Male vs female	0.35	0.03	NA	NA
f-Hb, μg Hb/g				
Undetected	−0.35	0.03	10.25	NA
1-9	0	NA	14.55	NA
10-19	0.12	0.04	16.38	NA
20-49	0.75	0.05	30.71	30.2
50-99	1.26	0.07	51.23	NA
100-149	1.27	0.08	51.65	NA
≥150	2.29	0.04	143.64	NA

Abbreviations: f-Hb, fecal-hemoglobin; NA, not applicable.

^a^ The mortality rate in the general population aged 50 to 74 years was obtained from the Taiwanese Cancer Registry.

**Table 3.** Relative Risk of CRC Death and Precision Interscreening Intervals Determined by f-Hb

f-Hb, μg Hb/g	Relative risk	Screening interval, y	
		Theoretical recommendation	Pragmatic recommendation
Undetected	0.33	6.00	6
1-9	0.47	4.22	4
10-19	0.53	3.75	3
20-49	1.00	2.00	2
50-99	1.67	1.20	1
100-149	1.68	1.19	1
≥150	4.68	0.43	0.5

Abbreviations: CRC, colorectal cancer; f-Hb, fecal-hemoglobin.

### Comparisons of Utilizations in FIT Tests and Colonoscopies Between Universal and f-Hb–Guided Personalized Screening Regimes

Under the context of universal and personalized screening strategies as shown in the Figure, a total of 19 917 993 were obtained from the clinical data and 10 233 766 FIT tests were imputed as outlined in Table 4. These data underscore the potential advantages of an f-Hb–guided personalized screening approach over universal screening. Specifically, the use of FIT tests decreased by 49% and colonoscopies by 28% given 80% referral rate, if personalized f-Hb–guided screening were adopted instead of the universal biennial screening strategy. This corresponding figure ranged from 11% to 46% when the referral rate varied from 100% to 60% in f-Hb–guided personalized screening.

**Table 4.** Number of FIT Tests, and Number of Colonoscopies for f-Hb–Guided Interscreening Intervals Compared With Universal Screening in 3 487 559 Participants

Variable	No.			
	Universal biennial screening	f-Hb–guided personalized CRC screening		
		60% Referral rate	80% Referral rate	100% Referral rate
Tests	19 917 993	10 233 766	NA	NA
Reduction in tests	1 [Reference]	9 684 228	NA	NA
Reduction in tests, %	1 [Reference]	49	NA	NA
Colonoscopies	1 279 680	686 570	915 427	1 144 284
Reduction in colonoscopies	1 [Reference]	593 110	364 253	135 396
Reduction in colonoscopies, %	1 [Reference]	46	28	11

Abbreviations: CRC, colorectal cancer; f-Hb, fecal-hemoglobin; FIT, fecal immunological test; NA, not applicable.

## Discussion

Within the contemporary landscape of population-based CRC screening employing FIT, a comprehensive evaluation of the intricate interplay between f-Hb and CRC related outcomes is warranted. Leveraging a robust foundation derived from a large screening cohort, this study has effectively unveiled the gradient relationship between f-Hb concentration and both colorectal neoplasia and its mortality. The empirical underpinnings of these findings not only support the role of FIT screening for both primary prevention of arresting incident CRC through the removal of adenoma by colonoscopy and the early detection of invasive CRC but also provide the plausible avenue for the formulation of personalized CRC screening using FIT.^19,20^ As mentioned in previous studies, risk stratification of the underlying population based on f-Hb categories facilitates surveillance of the high-risk group with intensive follow-up and repeated screening of the low-risk group with longer screen intervals.^13,16,21,22^

The preceding evidence suggests that information on an individual’s f-Hb can be used more effectively for multiple purposes, such as the possibility of conducting individualized screening.^16^ In the Netherlands, preparations are underway for a trial to explore the potential benefits of personalized screening intervals based on the prior f-Hb concentration.^23^ Moreover, quantitative FIT could potentially serve as a guide for determining optimal surveillance intervals in colonoscopy screening. Winter et al^24^ initiated a trial, implementing FIT at 3-year or 5-year surveillance intervals, compared with a control group where no FIT was required. The objective was to assess whether tailoring the frequency of surveillance colonoscopies through personalized FIT-based approaches could reduce unnecessary colonoscopies. Our study substantiates incremental variations in f-Hb concentrations as prudent stratification factors, allowing the at-risk population to be divided into discrete risk strata. Individuals manifesting considerably heightened f-Hb levels may necessitate shorten interscreening intervals, potentially involving FIT testing or expedited recourse to direct colonoscopy during subsequent screening initiatives. Conversely, individuals evincing lower f-Hb levels could be accorded prolonged intervals between FIT tests or colonoscopy screenings. The assimilation of this personalized screening paradigm not only averts instances of false-positive CRC diagnoses within the low-risk demographic for reduction of anxiety of patients, courtesy of elongated screening intervals, but also increase the chances of preventing CRC in high-risk patients via the frequent FIT measurements or the provision of advanced medical care. Because the majority of the population falls below the average risk level, this stance clearly explains the evident rationale behind the discernible reductions of 49% in FIT tests and 28% in colonoscopies, consequent to the assimilation of a personalized screening regimen, as contrasted against a universal screening modality. The comparative analysis of FIT test and colonoscopy use under the circumstance of equal efficacy would indirectly make inference about the minimization of cost due to less use of test and colonoscopy while using cost minimization design in cost-effectiveness analysis.^25^

Because the referral rate may be changed in the personalized screening setting, we also conducted a sensitivity analysis to assess the effect of referral on the colonoscopy reduction. A higher referral rate resulted in a lower reduction of colonoscopies, whereas a lower referral rate would lead to a greater reduction. Because referral rate has been associated with the effectiveness of screening, further investigation is required in the form of a randomized clinical trial to ascertain if the adherence to colonoscopy would be influenced by the implementation of personalized f-Hb–guided screening. In any case, the advantages of personalized f-Hb–guided screening was not only associated with a reduction of FIT and colonoscopies but also mitigated the potential risk of overdetection in low-risk groups.

The baseline f-Hb concentration and the underlying incidence and mortality of CRC for the average-risk group may vary across different populations. Therefore, although this methodology for designing precision interscreening intervals could be applied to other populations, the calibrations are required. There are different methods for such a calibration. In Dutch PERFECT-FIT trial,^23^ for example, the personalized screen intervals were targeted at the negative findings who had the prior FIT under a cut of 47 μg/g and determined by a priori knowledge derived from the previous well-conducted studies. For the intervention group, the 2-year interval as control group was set for greater than 0 to 15 μg/g, the short interval was set as 1 year for greater than 15 to 46 μg/g, but the longer interval was set as 3 years for 0 μg/g.^23^ Given our current findings, however, before implementation of a precision interscreening interval policy at large scale, a randomized clinical trial with 2 arms, the personalized screening arm and the universal screening arm, should be further proposed to test the feasibility in the light of the baseline f-HB categories particularly from the perspective of shared decision-making between clinical professionals and patients.

### Strengths and Limitations

The strengths of this study include the large sample of participants participating in FIT screening for whom both quantitative FIT results and outcomes with longitudinal follow-up were available. However, there are some limitations in the study. The analysis did not include the additional information on other risk factors associated with CRC. Although using f-Hb concentrations could offer a straightforward method to personalize screening, several studies have suggested incorporating additional individual characteristics—such as family history, body mass index, and smoking—as risk scores in conjunction with FIT to enhance the precision of cancer detection.^26,27^ There are already systems in place where information such as sex, age, and the quantitative FIT result is registered in existing databases for all screening participants. Additional information on risk factors associated with CRC is not routinely collected in our FIT screening program. However, the screening intervals for various risk stratifications could be adapted in the analysis if more comprehensive individual information becomes available.

## Conclusion

The findings of this study not only confirmed the previous gradient relationship between f-Hb levels and incidence and mortality on which we have based distinct proposals for personalized screening paradigms. Using f-Hb, we demonstrated how to achieve precision interscreening interval of population-based FIT screening for optimizing the use of FIT and colonoscopies.

## Notes

Supplement 1. eFigure 1. Cumulative advanced colorectal cancer (Stage ≥3) incidence by baseline fecal hemoglobin concentration eFigure 2. Cumulative colorectal cancer mortality by baseline fecal hemoglobin concentration eTable 1. Relative risk of advanced cancer and precision inter-screening intervals determined by f-Hb eTable 2. Relative risk of interval cancer and precision inter-screening intervals determined by f-Hb

Supplement 2. Data Sharing Statement
