# Supplementary material for: Precision Colorectal Cancer Fecal Immunological Test Screening With Fecal-Hemoglobin-Concentration–Guided Interscreening Intervals
Source: JAMA Oncol. 2024 May 9;10(6):765–72. doi: 10.1001/jamaoncol.2024.0961 (PMC11082752; doi:10.1001/jamaoncol.2024.0961)
Supplement: Supplement 1. — eFigure 1. Cumulative advanced colorectal cancer (Stage ≥3) incidence by baseline fecal hemoglobin concentration eFigure 2. Cumulative colorectal cancer mortality by baseline fecal hemoglobin concentration eTable 1. Relative risk of advanced cancer and precision inter-screening intervals determined by f-Hb eTable 2. Relative risk of interval cancer and precision inter-screening intervals determined by f-Hb [file jamaoncol-e240961-s001.pdf]

Supplemental Online Content

Yen A M-F, Hsu C-Y, Lin T-Y, et al. Precision colorectal cancer fecal immunological test screening with fecal-hemoglobin centration–guided interscreening intervals. *JAMA Oncol*. Published online May 9, 2024.
doi:10.1001/jamaoncol.2024.0961

eFigure 1. Cumulative advanced colorectal cancer (Stage ≥3) incidence by baseline fecal hemoglobin concentration
eFigure 2. Cumulative colorectal cancer mortality by baseline fecal hemoglobin concentration
eTable 1. Relative risk of advanced cancer and precision inter-screening intervals determined by f-Hb
eTable 2. Relative risk of interval cancer and precision inter-screening intervals determined by f-Hb

This supplemental material has been provided by the authors to give readers additional information about their work.

© 2024 American Medical Association. All rights reserved.

eFigure 1. Cumulative advanced colorectal cancer (Stage>=III) incidence by baseline fecal hemoglobin concentration.

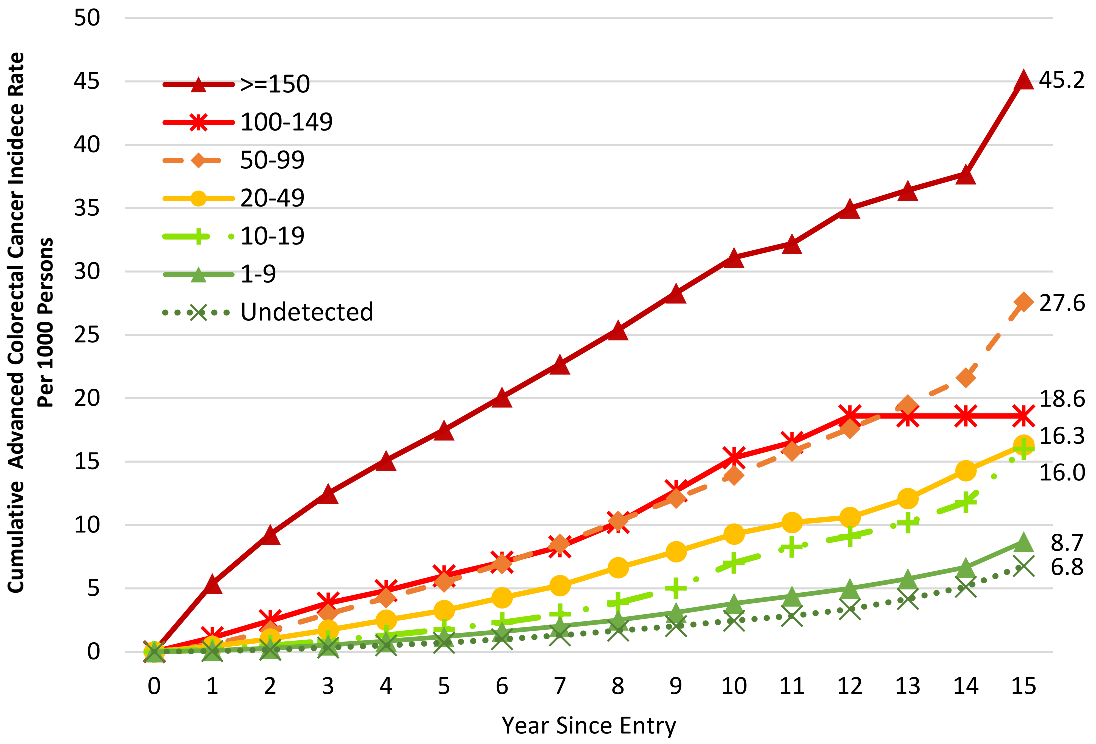

© 2024 American Medical Association. All rights reserved.

eFigure 2. Cumulative colorectal cancer mortality by baseline fecal hemoglobin concentration.

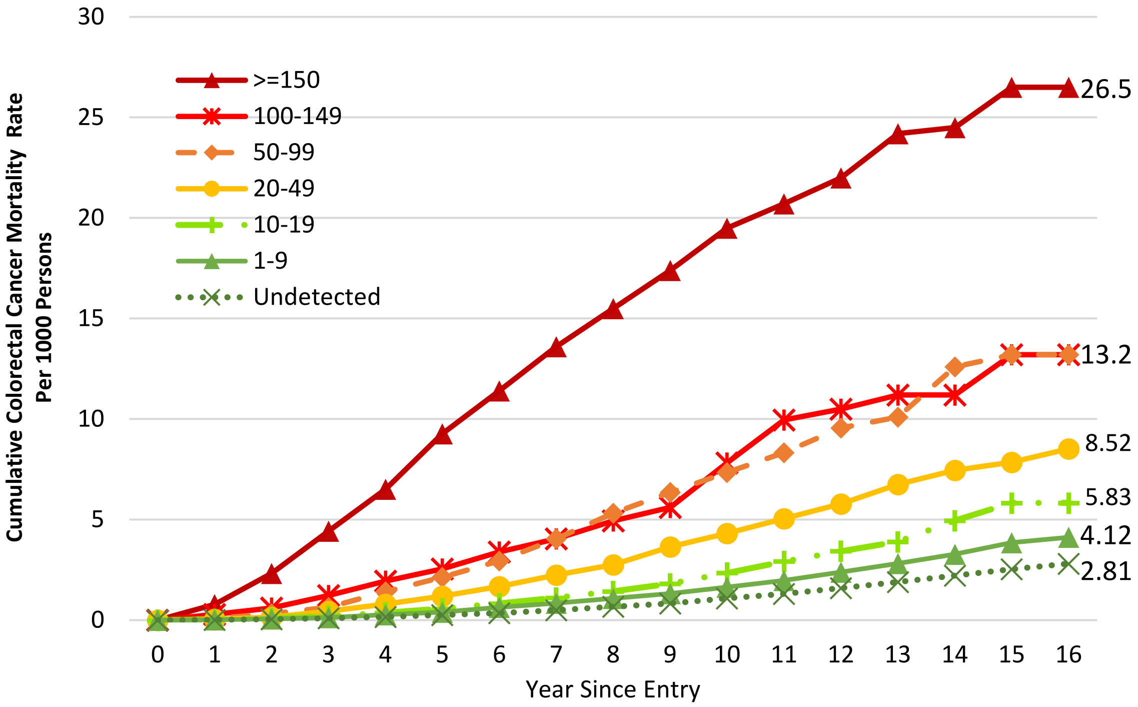

© 2024 American Medical Association. All rights reserved.

eTable 1. Relative risk of advanced cancer and precision inter-screening intervals determined by f-Hb

f-Hb (μg Hb/g)	Observed Advanced Cancer Rate (Per 100,000)	Estimated Advanced Cancer Rate (Per 100,000)	Relative Risk	Screening Interval (year)	
				Theoretical Recommendation	Pragmatic Recommendation
Undetected	23.90	23.24	0.32	6.17	6
1-9	35.00	32.88	0.46	4.36	4
10-19	47.10	43.85	0.61	3.27	3
20-49	80.74	71.67	1.00	2.00	2
50-99	128.43	111.32	1.55	1.29	1.0
100-149	127.81	113.16	1.58	1.27	1.0
>=150	328.03	280.78	3.92	0.51	0.5

© 2024 American Medical Association. All rights reserved.

eTable 2: Relative risk of interval cancer and precision inter-screening intervals determined by f-Hb

f-Hb (μg Hb/g)	Observed Interval Cancer Rate (Per 100,000)	Estimated Interval Cancer Rate (Per 100,000)	Relative Risk	Screening Interval (year)	
				Theoretical Recommendation	Pragmatic Recommendation
Undetected	6.75	6.33	0.19	10.50	10
1-9	12.67	11.32	0.34	5.87	6
10-19	24.43	21.40	0.64	3.11	3
20-49	40.37	33.22	1.00	2.00	2
50-99	69.27	55.11	1.66	1.21	1.0
100-149	77.98	63.72	1.92	1.04	1.0
>=150	223.78	174.44	5.25	0.38	0.5

© 2024 American Medical Association. All rights reserved.
