# Supplementary material for: Precision Colorectal Cancer Fecal Immunological Test Screening With Fecal-Hemoglobin-Concentration–Guided Interscreening Intervals
Source: JAMA Oncol. 2024 May 9;10(6):765–72. doi: 10.1001/jamaoncol.2024.0961 (PMC11082752; doi:10.1001/jamaoncol.2024.0961)
Supplement: Supplement 2. — Data Sharing Statement [file jamaoncol-e240961-s002.pdf]

Data Sharing Statement

Yen. Precision Colorectal Cancer Fecal Immunological Test Screening with Fecal-Hemoglobin-Concentration–Guided Interscreening Intervals. *JAMA Oncol*. Published May 09, 2024. doi:10.1001/jamaoncol.2024.0961

Data
Data available: No
